# Monocyte-to-HDL cholesterol ratio and uric acid-to-HDL cholesterol ratio as predictors of vitamin D deficiency in healthy young adults: a cross-sectional study

**DOI:** 10.20945/2359-4292-2024-0004

**Published:** 2024-08-27

**Authors:** Aziz Sener, Semra Isikoglu Hatil, Elif Erdogan, Elif Durukan, Canan Topcuoglu

**Affiliations:** 1 Department of Medical Biochemistry Ankara Etlik City Hospital Türkiye Department of Medical Biochemistry, Ankara Etlik City Hospital, Türkiye; 2 Department of Public Health Baskent University Medicine Faculty Türkiye Department of Public Health, Baskent University Medicine Faculty, Türkiye

**Keywords:** Inflammation, vitamin D deficiency, monocyte-to-high-density lipoprotein cholesterol ratio, young adult, uric acid-to-high-density lipoprotein cholesterol ratio

## Abstract

**Objective:**

This study aimed to assess vitamin D deficiency in a cohort of healthy young adults using the novel inflammatory parameters neutrophil-to-lymphocyte ratio (NLR), platelet-to-lymphocyte ratio (PLR), monocyte-to-lymphocyte ratio (MLR), monocyte-to-high-density lipoprotein (HDL) cholesterol ratio (MHR), and uric acid-to-HDL cholesterol ratio (UHR).

**Subjects and methods:**

The study included 1,190 participants, with demographic and laboratory data retrieved retrospectively from our institution’s database. The inclusion criteria were ages 22-35 years; absence of acute, subacute, or chronic diseases; no regular medication use; and laboratory values within specified reference ranges. The exclusion criteria were pregnancy, diagnosis of malignancy, and laboratory measurements indicating infection. Participants were categorized into four groups based on vitamin D levels for comparative analysis of study parameters. Correlation analyses were conducted between these parameters and 25-hydroxyvitamin D levels, followed by receiver-operating characteristic analyses to determine the parameters’ sensitivity and specificity in detecting vitamin D deficiency. Additionally, regression analyses were performed to identify potential risk factors for vitamin D deficiency.

**Results:**

Subjects in Groups A1 and A2 exhibited higher MHR and UHR than those in Groups A3 and A4 (p < 0.001). Both MHR and UHR correlated negatively with vitamin D levels (r = -0.377 and r = -0.363, respectively; p < 0.001). The area under the curve for MHR was 0.766 (95% confidence interval [CI] 0.739-0.794), with a 79% sensitivity and 61% specificity for identification of vitamin D deficiency. Increased MHR and UHR were independent risk factors for vitamin D deficiency (β = 0.219, odds ratio 1.244 95% CI 1.178-1.315, and β = 2.202, odds ratio 1.224, 95% CI 1.150-1.303).

**Conclusions:**

Both MHR and UHR can be useful in predicting vitamin D deficiency in healthy young adults and may serve as valuable screening tools.

## INTRODUCTION

Vitamin D, a fat-soluble secosteroid, plays a crucial role in human health, encompassing various physiological functions beyond its classical role in calcium and phosphorus homeostasis (1). Vitamin D exerts its actions through two primary forms – vitamin D2 (ergocalciferol) and vitamin D3 (cholecalciferol) – acquired through ultraviolet B radiation from sunlight exposure, dietary intake, or supplementation (2). The active metabolite calcitriol regulates gene expression and influences immune modulation, cellular proliferation, and differentiation, affecting both skeletal health and immunomodulation and with potential links to non-skeletal health outcomes (3). The assessment, supplementation, and monitoring of vitamin D are long-standing topics of research and clinical significance in the fields of nutrition and medicine, given their correlation with manifold health concerns. Ensuring adequate vitamin D levels in young adulthood is essential for maintaining physical well-being, mental health, and a robust immune system. Vitamin D deficiency is anticipated in this age group owing to limited exposure to sunlight or insufficient dietary intake (4).

Vitamin D deficiency emerges as a potential contributor to dyslipidemia, a recognized risk factor for cardiovascular disease (5). Moreover, recent research indicates a significant association between low levels of vitamin D and unfavorable lipid alterations, characterized by elevated levels of total cholesterol (TC), low-density lipoprotein cholesterol (LDL-C), and triglycerides, with a concomitant reduction in high-density lipoprotein cholesterol (HDL-C) (6). These findings suggest a role for vitamin D in regulating lipid metabolism. Dyslipidemic conditions initiate inflammatory processes, underscoring the heightened significance of the regulatory role of vitamin D in lipid metabolism.

One reason vitamin D deficiency plays an important role in inflammation is the complex relationship between dyslipidemia and inflammation (7). Vitamin D also has immunomodulatory properties, which function with the innate and adaptive immune systems. Furthermore, vitamin D deficiency is associated with increased inflammation, as indicated by elevated levels of proinflammatory cytokines and markers of immune activation (8). The significance of the association between vitamin D deficiency and inflammation is unequivocal, particularly in autoimmune diseases, infectious diseases, and specific types of cancer (9-11). Therefore, attention to maintaining sufficient vitamin D levels is crucial in these contexts.

Vitamin D deficiency is characterized by heightened inflammatory responses in the body and is associated with inflammatory conditions (12). Derived from biochemical and complete blood count measurements, parameters like neutrophil-to-lymphocyte ratio (NLR), platelet-to-lymphocyte ratio (PLR), monocyte-to-lymphocyte ratio (MLR), uric acid-to-HDL-C ratio (UHR), and monocyte-to-HDL-C ratio (MHR) offer a straightforward, cost-effective means of inflammatory assessment. Recent studies have highlighted MHR as a dependable inflammatory marker, while UHR has been implicated in various inflammation-related conditions such as hypertension and type 2 diabetes mellitus (13-15). Given the significance of these inflammatory markers, exploring their relationship with vitamin D deficiency is pertinent. Their correlation with chronic or subacute diseases suggests potential links to vitamin D deficiency.

The close and significant relationship between vitamin D deficiency and inflammation is critical to consider in terms of health outcomes, especially for young adults who are prone to vitamin D deficiency. In light of these considerations, the primary goal of this study was to investigate thoroughly the effect of vitamin D deficiency on lipid profiles and its connection to new inflammatory parameters (NLR, PLR, MLR, MHR, and UHR) in a population of healthy young adults. This study also aimed to assess the predictability of vitamin D deficiency using new inflammatory parameters, focusing on MHR and UHR in this age population. With the findings from this study, we aimed to identify specific interventions and preventative measures for mitigating the harmful effects of vitamin D deficiency and inflammation among this population.

## SUBJECTS AND METHODS

### Study population

This study employed a retrospective, cross-sectional design. Following approval from the ethics committee, a comprehensive review was conducted on the data pertaining to individuals aged between 22 and 35 years who underwent biochemical and complete blood count analyses at the biochemistry laboratory of Ankara Etlik City Hospital from January 1, 2023, to October 1, 2023. The participants’ demographic information, including age and sex, along with laboratory and clinical data were retrospectively extracted from the hospital’s automated system and patient records. Cohort selection was nonrandom, as all individuals meeting the predefined inclusion criteria within the specified date range were incorporated into the study cohort. The laboratory and demographic data of 1,190 individuals were included in the study.

The inclusion criteria were ages 22-35 years; absence of acute, subacute, or chronic diseases; no regular medication use; and laboratory values within the reference range for creatinine, albumin, total bilirubin, urea, and alanine aminotransferase (ALT). Additionally, comprehensive results pertaining to biochemical and complete blood count parameters relevant to our research were essential for consideration.

We excluded patients who were pregnant and those with a diagnosis of malignancy, laboratory measurements indicating infection (*i.e.*, elevated white blood cell count, lymphopenia, or neutrophilia), active complaints, active inflammatory conditions, advanced chronic diseases (*i.e.*, heart failure, chronic kidney disease, liver failure), missing data related to the study parameters, and missing clinical information. Our sample group consisted primarily of individuals who underwent routine health screenings or elective surgeries, such as rhinoplasty and aesthetic procedures at our hospital, and had no active complaints.

### Data collection and laboratory measurements

We collected the participants’ demographic data, including age and sex, alongside an extensive range of laboratory measurements. These measurements encompassed creatinine, urea, uric acid, calcium, total bilirubin, ALT, albumin, TC, HDL-C, LDL-C, triglycerides, 25-hydroxyvitamin D (25[OH]D), and complete blood count parameters.

The following calculations were made:

- NLR = absolute neutrophil count ÷ lymphocyte count
- PLR = platelet count ÷ lymphocyte count
- MLR = monocyte count ÷ lymphocyte count
- MHR = monocyte count ÷ HDL-C level
- UHR = uric acid level ÷ HDL-C level
- TC/HDL-C = TC level ÷ HDL-C level
- LDL-C/HDL-C = LDL-C level ÷ HDL-C level

Uric acid, creatinine, urea, total bilirubin, albumin, ALT, calcium, triglycerides, TC, and HDL-C were analyzed by spectrophotometry using the Cobas c702 system (Roche, Germany). Levels of 25(OH)D were analyzed by competitive immunoassay using the Cobas e801 system (Roche, Germany). Complete blood count parameters were analyzed using the Sysmex XN-1000 analyzer (Kobe, Japan).

### Groups and definition of cutoff points

Groups according to vitamin D levels were formed based on the guidelines for the Diagnosis and Treatment of Osteoporosis and Metabolic Bone Diseases of the Society of Endocrinology and Metabolism of Türkiye (16). The groups were formed according to 25(OH)D levels: Group A1, 25(OH)D level < 10 ng/mL (25 nmol/L); Group A2, 10 ng/mL (25 nmol/L) ≤ 25(OH)D level ≤ 20 ng/mL (50 nmol/L); Group A3, 20 ng/mL (50 nmol/L) < 25(OH)D level ≤ 30 ng/mL (75 nmol/L); and Group A4, 25(OH)D level > 30 ng/mL (75 nmol/L). The 25(OH)D level of 20 ng/mL (50 nmol/L) was used as a cutoff to define vitamin D deficiency for receiver-operating characteristic (ROC) and regression analyses.

### Statistical analysis

The normality assumption for continuous data was assessed using the Kolmogorov-Smirnov test, skewness, and kurtosis. Group comparisons were conducted using the analysis of variance (ANOVA) test for variables with a normal distribution, while those without a normal distribution were analyzed with the Kruskal-Wallis test. After these analyses, the *post hoc* Tukey test was used to discern group differences for normally distributed parameters. Conversely, the Mann-Whitney U test was used for non-normally distributed parameters. Sex distribution among groups was assessed using the chi-square test. Spearman’s correlation test was applied to investigate the association between 25(OH)D and other parameters. The diagnostic and screening accuracy of the parameters were determined by constructing ROC curves. Univariate logistic regression analysis was performed to identify potential predictors of vitamin D deficiency and to build a multivariate logistic regression model. The vitamin D deficiency group was labeled as 1, while the group with normal vitamin D levels was labeled as 0. Parameters displaying a significance level < 0.1 in the univariate logistic regression analysis were included in subsequent multivariate logistic regression analysis. Variables highly correlated with MHR, UHR, and NLR (uric acid, TC, LDL-C, HDL-C, lymphocyte count, monocyte count, and MLR) were intentionally excluded from the multivariate logistic regression analysis to mitigate multicollinearity. Variables with and without normal distribution are expressed as mean ± standard deviation and median (interquartile range), respectively. A p value lower than 0.05 was considered statistically significant. Statistical analyses were conducted using the software SPSS, Version 25.0 (SPSS Inc., Chicago, IL, USA).

### Ethical considerations

Ethical approval for our study was granted by the Ethics Committee of Ankara Etlik City Hospital (Decision number, AEŞH-EK1-2023-648; Date, November 1, 2023). The study strictly adhered to the Declaration of Helsinki. Informed consent was not obtained from the individuals, as the nature of the study entailed a retrospective examination of deidentified blood data, and no specific biological samples were procured from the individuals in this investigation.

## RESULTS

**Comparison of groups**

**Figure 1:**
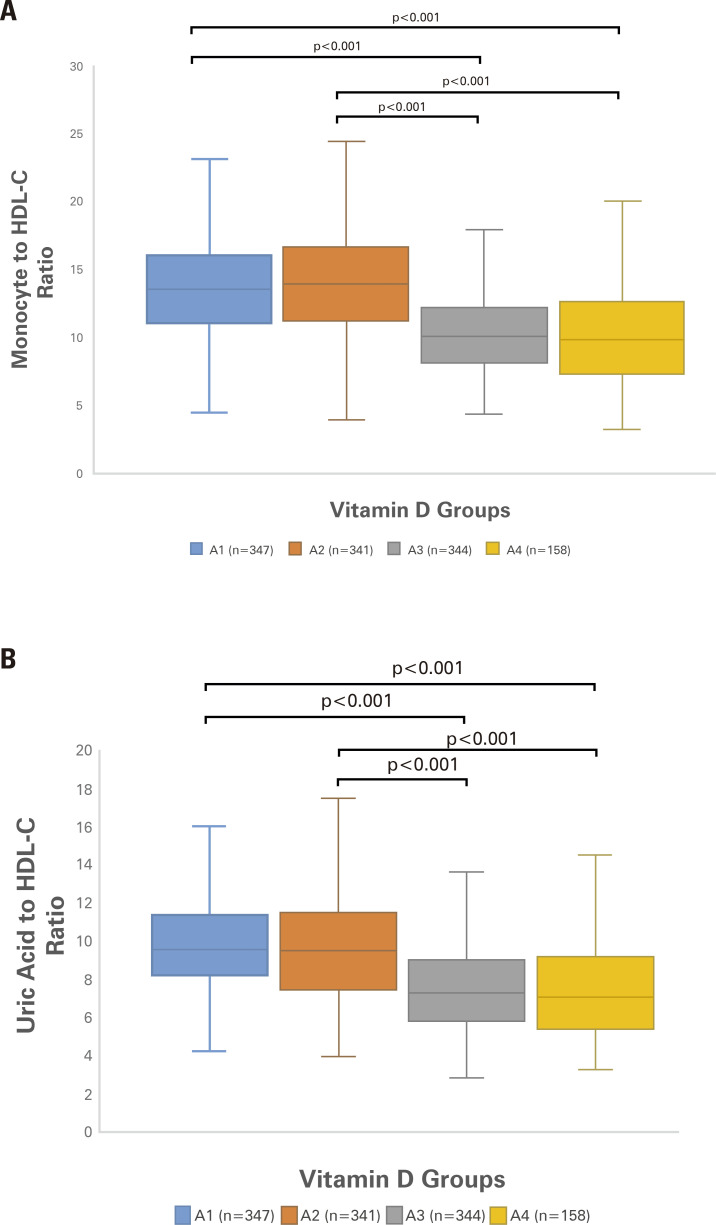
Distribution of (A) monocyte-to-high-density lipoprotein cholesterol ratio (MHR) and (B) uric acid-to-high-density lipoprotein cholesterol ratio (UHR) parameters among the groups. Both MHR and UHR varied among the groups (p < 0.001 for each) and were higher in Groups A1 and A2 subjects than in Groups A3 and A4 subjects (p < 0.001 for each). Abbreviation: HDL-C, high-density lipoprotein cholesterol.

Demographic characteristics and laboratory measurements of the groups formed according to vitamin D levels are depicted in Table 1. As shown, MHR, UHR, TC/HDL-C, and LDL-C/HDL-C varied among the groups (p < 0.001 for each) and were higher in subjects in Groups A1 and A2 than in those in Groups A3 and A4 (p < 0.001 for each) (Figure 1).

**Table 1:** Demographic characteristics and laboratory results of the groups divided according to vitamin D levels

Parameters	Group A1 (D < 10 ng/mL; n = 347)	Group A2 (10 ng/mL ≤ D ≤ 20 ng/mL; n = 341)	Group A3 (20 ng/mL < D ≤ 30 ng/mL; n = 344)	Group A4 (D > 30 ng/mL; n = 158)	P values
Sex (n, female/male)	293/54	178/163	267/77	117/41	**<0.001**^**c**^
Age (years)	27.86 ± 3.98	28.50 ± 3.94	28.49 ± 4.03	29.18 ± 4.08	**<0.05**^**a**^
Uric acid (mg/dL)	4.39 ± 0.92	4.23 ± 0.99	4.04 ± 0.90	3.97 ± 0.95	**<0.001**^**a**^
Urea (mg/dL)	27.64 ± 6.25	27.23 ± 6.27	26.76 ± 6.25	27.50 ± 6.59	>0.05^a^
Creatinine (mg/dL)	0.74 ± 0.13	0.75 ± 0.13	0.74 ± 0.15	0.75 ± 0.17	>0.05^a^
Total bilirubin (mg/dL)	0.73 ± 0.28	0.70 ± 0.29	0.70 ± 0.29	0.67 ± 0.30	>0.05^a^
ALT (U/L)	17.47 ± 9.30	18.54 ± 9.56	16.78 ± 9.31	16.92 ± 8.94	>0.05^a^
Calcium (mg/dL)	9.46 ± 0.38	9.49 ± 0.34	9.47 ± 0.37	9.49 ± 0.34	>0.05^a^
Albumin (g/L)	43.62 ± 3.50	43.77 ± 3.55	43.95 ± 3.48	44.06 ± 3.73	>0.05^a^
Triglyceride (mg/dL)	110.80 ± 39.23	114.14 ± 39.98	110.23 ± 40.04	113.24 ± 40.92	>0.05^a^
TC (mg/dL)	154.79 ± 22.21	153.47 ± 22.12	156.90 ± 22.02	158.92 ± 26.97	>0.05^a^
LDL-C (mg/dL)	87.05 ± 20.67	85.53 ± 19.05	78.99 ± 17.74	79.99 ± 21.82	**<0.001**^**a**^
HDL-C (mg/dL)	45.61 ± 10.18	45.12 ± 10.77	55.89 ± 13.58	56.30 ± 16.46	**<0.001**^**a**^
25(OH)D (ng/mL)	7 (6-8)	15 (13-17)	24 (21-26)	35 (32-41)	**<0.001**^**k**^
WBC (×10^3^/µL)	7.81 ± 1.05	7.73 ± 1.06	7.68 ± 1.04	7.45 ± 1.00	**<0.05**^**a**^
Neutrophil (×10^3^/µL)	4.43 ± 0.88	4.35 ± 0.93	4.53 ± 0.99	4.34 ± 0.92	**<0.05**^**a**^
Lymphocyte (×10^3^/µL)	2.53 ± 0.58	2.52 ± 0.59	2.40 ± 0.59	2.37 ± 0.60	**<0.01**^**a**^
Monocyte (×10^3^/µL)	0.60 ± 0.13	0.61 ± 0.14	0.55 ± 0.13	0.55 ± 0.13	**<0.001**^**a**^
Hemoglobin (g/dL)	13.62 ± 1.63	13.78 ± 2.24	13.84 ± 1.46	13.95 ± 1.56	>0.05^a^
PLT (x10^3^/µL)	314 (282-353)	264 (232-312)	283 (253-329)	282.5 (245-323)	**<0.001**^**k**^
MPV (fL)	10.33 ± 0.83	10.43 ± 0.88	10.40 ± 0.87	10.53 ± 0.90	>0.05^a^
RDW (%)	13.2 (12.6-14.6)	12.7 (12.3-13.3)	13 (12.4-13.6)	12.9 (12.3-13.5)	**<0.001**^**k**^
IG (×10^3^/µL)	0.02 (0.02-0.03)	0.02 (0.02-0.03)	0.02 (0.01-0.03)	0.02 (0.01-0.03)	**<0.001**^**k**^
NLR (%)	1.72 (1.40-2.21)	1.68 (1.33-2.20)	1.85 (1.48-2.34)	1.80 (1.43-2.40)	**<0.05**^**k**^
PLR (%)	126.78 (107.65-155.29)	110.41 (90.49-133.40)	124.36 (101.77-147.21)	119.12 (100.04-146.12)	**<0.001**^**k**^
MLR (%)	0.243 (0.199-0.287)	0.237 (0.199-0.285)	0.222 (0.181-0.274)	0.228 (0.192–0.279)	**<0.01**^**k**^
MHR (%)	13.95 ± 4.19	14.13 ± 3.98	10.21 ± 2.89	10.78 ± 4.76	**<0.001**^**a**^
UHR (%)	9.98 ± 2.58	9.91 ± 3.39	7.63 ± 2.57	7.66 ± 3.04	**<0.001**^**a**^
TC/HDL-C	3.54 ± 0.89	3.54 ± 0.80	2.93 ± 0.63	3.00 ± 0.78	**<0.001**^**a**^
LDL-C/HDL-C	2.04 ± 0.79	2.01 ± 0.69	1.51 ± 0.53	1.56 ± 0.64	**<0.001**^**a**^

^a^ Analysis of variance (ANOVA) test. ^c^ Chi-square test. ^K^ Kruskal-Wallis test. Values are shown as mean ± standard deviation or median (interquartile range). P values < 0.05 (shown in bold) were considered statistically significant. Abbreviations: 25(H)D, 25-hydroxyvitamin D; ALT, alanine transaminase; D, 25-hydroxyvitamin D level; HDL-C, high-density lipoprotein cholesterol; IG, immature granulocyte count; LDL-C/HDL-C, low-density lipoprotein cholesterol-to-high-density lipoprotein cholesterol ratio; LDL-C, low-density lipoprotein cholesterol; MHR, monocyte-to-high-density lipoprotein cholesterol ratio; MLR, monocyte-to-lymphocyte ratio; MPV, mean platelet volume; NLR, neutrophil-to-lymphocyte ratio; PLR, platelet-to-lymphocyte ratio; PLT, platelet count; RDW, red cell distribution width; TC, total cholesterol; TC/HDL-C, total cholesterol-to-high-density lipoprotein cholesterol ratio; UHR, uric acid-to-high-density lipoprotein cholesterol ratio; WBC, white blood cell count.

### Correlation status of the parameters with vitamin D levels

Monocyte (r = -0.206), uric acid (r = -0.137), LDL-C (r = -0.142), MHR (r = -0.377), UHR (r = -0.363), LDL-C/HDL-C (r = -0.295), and TC/HDL-C (r = -0.299) correlated negatively with vitamin D levels (p < 0.001 for each). Additionally, HDL-C correlated positively with vitamin D levels (r = 0.321, p < 0.001) (Table 2).

**Table 2:** Correlation status of the analyzed parameters with 25-hydroxyvitamin D (25[OH]D) vitamin levels

Parameters	R values	P values
Uric Acid (mg/dL)	-0.137	**<0.001**
LDL-C (mg/dL)	-0.142	**<0.001**
HDL-C (mg/dL)	0.321	**<0.001**
Neutrophil (×10^3^/µL)	-0.009	>0.05
Lymphocyte (×10^3^/µL)	-0.102	**<0.001**
Monocyte (×10^3^/µL)	-0.206	**<0.001**
RDW (%)	-0.127	**<0.001**
MPV (fL)	0.064	**<0.05**
NLR	0.061	**<0.05**
PLR	-0.070	**<0.05**
MLR	-0.084	**<0.01**
MHR	-0.377	**<0.001**
UHR (%)	-0.363	**<0.001**
TC/HDL-C	-0.299	**<0.001**
LDL-C/HDL-C	-0.295	**<0.001**

The correlation between 25(OH)D levels and the analyzed parameters was evaluated using Spearman’s Rho test. P values < 0.05 (shown in bold) were considered statistically significant. Abbreviations: HDL-C, high-density lipoprotein cholesterol; LDL-C, low-density lipoprotein cholesterol; LDL-C/HDL-C, low-density lipoprotein cholesterol-to-high-density lipoprotein cholesterol ratio; MHR, monocyte-to-high-density lipoprotein cholesterol ratio; MLR, monocyte-to-lymphocyte ratio; MPV, mean platelet volume; NLR, neutrophil-to-lymphocyte ratio; PLR, platelet-to-lymphocyte ratio; TC/HDL-C, total cholesterol-to-high-density lipoprotein cholesterol ratio; UHR, uric acid-to-high-density lipoprotein cholesterol ratio; R, correlation coefficient; RDW, red cell distribution width.

### Vitamin D deficiency: associated factors

Multivariate logistic regression analysis was conducted to assess the predictive factors for vitamin D deficiency, incorporating sex, age, ALT, hemoglobin, platelet count, red cell distribution width (RDW), NLR, MHR, UHR, white blood cell count (WBC), and LDL-C/HDL-C as potential predictors. The findings revealed that men were more susceptible to vitamin D deficiency than women (β = 0.407; p = 0.035). Additionally, reduced hemoglobin level emerged as a risk factor for vitamin D deficiency (β = -0.306, p < 0.001). Lastly, elevated MHR and UHR were established as independent risk factors for vitamin D deficiency (β = 0.219, p < 0.001 and β = 2.202, p < 0.001, respectively) (Table 3).

**Table 3:** Regression analysis for identification of predictors of vitamin D deficiency

Variables	Univariate logistic regression analysis		Multivariate logistic regression analysis	
	β/OR (95% CI)	P values	β/OR (95% CI)	P values
Sex	0.315/0.485 (0.375-0.627)	**<0.001**	0.407/1.503 (1.030-2.193)	0.035
Age	-0.033/0.968 (0.940-0.996)	**0.025**	-0.048/0.953 (0.921-0.987)	**0.007**
Uric acid (mg/dL)	0.337/1.401 (1.235-1.589)	**<0.001**	-	
Urea (mg/dL)	0.011/1.011 (0.993-1.030)	0.233	-	
Creatinine (mg/dL)	0.310/1.364 (0.609-3.054)	0.450	-	
Total bilirubin (mg/dL)	0.304/1.355 (0.909-2.021)	0.136	-	
ALT (U/L)	0.014/1.014 (1.001-1.027)	**0.032**	-0.017/0.983 (0.966-1.000)	0.058
Calcium (mg/dL)	-0.002/0.998 (0.725-1.375)	0.992	-	
Albumin (g/L)	-0.023/0.977 (0.946-1.010)	0.165	-	
Triglycerides (mg/dL)	0.001/1.001 (0.998-1.004)	0.585	-	
Total cholesterol (mg/dL)	-0.007/0.993 (0.988-0.999)	**0.011**	-	
LDL-C (mg/dL)	0.018/1.018 (1.012-1.025)	**<0.001**	-	
HDL-C (mg/dL)	-0.069/0.933 (0.923-0.943)	**<0.001**	-	
WBC (×10^3^/µL)	0.148/1.160 (1.038-1.296)	**0.009**	-0.057/0.944 (0.812-1.098)	0.457
Neutrophil (×10^3^/µL)	-0.089/0.914 (0.809-1.034)	0.154	-	
Lymphocyte (×10^3^/µL)	0.385/1.469 (1.202-1.795)	**<0.001**	-	
Monocyte (×10^3^/µL)	3.674/39.424 (15.55-99.911)	**<0.001**	-	
Hemoglobin (g/dL)	-0.056/0.945 (0.886-1.009)	0.089	-0.306/0.737 (0.665-0.816)	**<0.001**
Platelet (×10^3^/µL)	0.003/1.003 (1.001-1.005)	**0.003**	0.004/1.004 (1.001-1.006)	**0.003**
MPV (fL)	-0.087/0.917 (0.803-1.047)	0.198	-	
RDW (%)	0.122/1.130 (1.041-1.226)	**0.003**	0.002/1.002 (0.903-1.112)	0.967
NLR (%)	-0.234/0.791 (0.688-0.909)	**<0.001**	-0.136/0.873 (0.743-1.025)	0.098
PLR (%)	-0.001/0.999 (0.997-1.002)	0.489	-	
MLR (%)	1.324/3.760 (0.960-14.725)	0.057	-	
MHR (%)	0.268/1.307 (1.257-1.360)	**<0.001**	0.219/1.244 (1.178-1.315)	**<0.001**
UHR (%)	0.308/1.360 (1.292-1.431)	**<0.001**	2.202/1.224 (1.150-1.303)	**<0.001**
TC/HDL-C	1.070/2.917 (2.426-3.506)	**<0.001**	-	
LDL-C/HDL-C	1.233/3.430 (2.764-4.257)	**<0.001**	0.271/1.311 (0.980-1.752)	0.068

Univariate logistic regression analysis was employed to identify potentially significant predictors of vitamin D deficiency, where the group with vitamin D deficiency served as the dependent variable denoted as 1, while the group with normal vitamin D levels was labeled 0. Parameters exhibiting a significance level < 0.1 in the univariate logistic regression analysis were incorporated into the subsequent multivariate logistic regression analysis. To mitigate the risk of multicollinearity, parameters displaying high correlation with MHR and UHR (uric acid, total cholesterol, LDL-C, HDL-C, lymphocyte count, and monocyte count) were deliberately excluded from the multivariate logistic regression analysis. Statistically significant parameters are shown in bold. Abbreviations: ALT, alanine transaminase; β, beta coefficient; CI, confidence interval; HDL-C, high-density lipoprotein cholesterol; LDL-C, low-density lipoprotein cholesterol; LDL-C/HDL-C, low-density lipoprotein cholesterol-to-high-density lipoprotein cholesterol ratio; MHR, monocyte-to-high-density lipoprotein cholesterol ratio; MLR, monocyte-to-lymphocyte ratio; MPV, mean platelet volume; NLR, neutrophil-to-lymphocyte ratio; PLR, platelet-to-lymphocyte ratio; OR, odds ratio; RDW, red cell distribution width; UHR, uric acid-to-high-density lipoprotein cholesterol ratio; TC/HDL-C, total cholesterol-to-high-density lipoprotein cholesterol ratio; WBC, white blood cell count.

### Receiver-operating characteristic analysis of parameters

**Figure 2:**
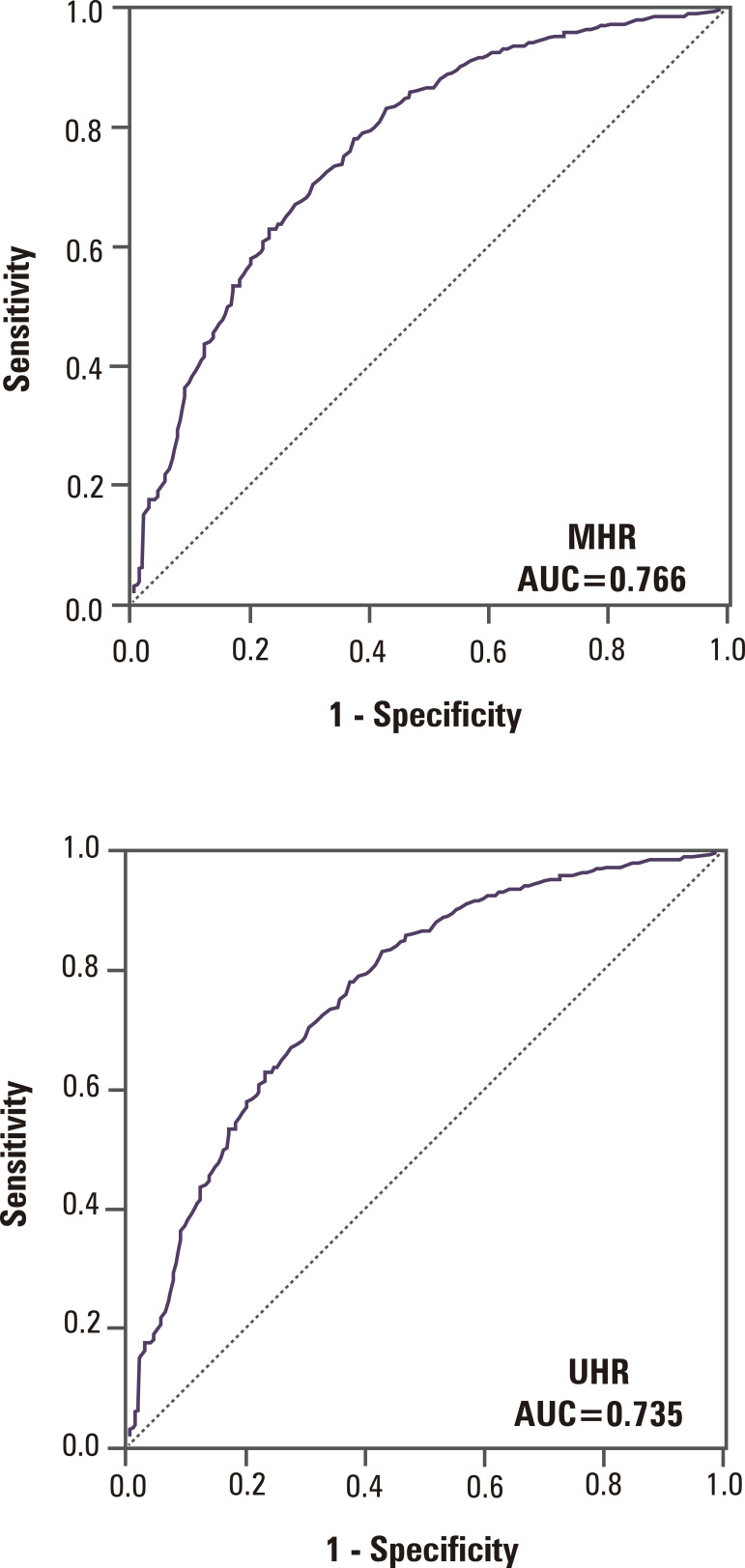
Diagnostic role of (A) monocyte-to-high-density lipoprotein cholesterol ratio (MHR) and (B) uric acid-to-high-density lipoprotein cholesterol ratio (UHR) parameters in vitamin D deficiency (below 20 ng/mL). The MHR cutoff value of 10.87 showed an area under the receiver-operating characteristic curve (AUC) of 0.766 (95% confidence interval [CI] 0.739-0.794) and 79% sensitivity and 61% specificity for vitamin D deficiency. The UHR cutoff value of 7.55 showed an AUC of 0.735 (95% CI 0.683-0.764) and 79% sensitivity and 55% specificity for vitamin D deficiency.

The results of ROC analysis of MHR, UHR, TC/HDL-C, and LDL-C/HDL-C are shown in Table 4. A cutoff of 20 ng/mL (50 nmol/L) was used to define vitamin D deficiency for the ROC analysis. A MHR cutoff value of 10.87 had an area under the curve (AUC) of 0.766 (95% confidence interval [CI] 0.739-0.794) with a sensitivity of 79% and specificity of 61% for identifying vitamin D deficiency (Figure 2). A UHR cutoff value of 7.55 had an AUC of 0.735 (95% CI 0.683-0.764) with a sensitivity of 79% and specificity of 55% for identifying vitamin D deficiency.

**Table 4:** Receiver-operating characteristic (ROC) analysis and optimal cutoff values for MHR, UHR, TC/HDL-C, and LDL-C/HDL-C

Parameters	AUC (95% CI)	P values	Sensitivity (%)	Specificity (%)	Cutoff
MHR	0.766 (0.739-0.794)	<0.001	79	61	10.87
UHR (%)	0.735 (0.706-0.764)	<0.001	79	55	7.55
TC/HDL-C	0.712 (0.683-0.741)	<0.001	79	48	2.80
LDL-C/HDL-C	0.706 (0.676-0.735)	<0.001	79	48	1.40

Abbreviations: AUC, area under the receiver-operating characteristic curve; CI, confidence interval; LDL-C/HDL-C, low-density lipoprotein cholesterol-to-high-density lipoprotein cholesterol ratio; MHR, monocyte-to-high-density lipoprotein cholesterol ratio; ROC, receiver-operating characteristic; TC/HDL-C, total cholesterol-to-high-density lipoprotein cholesterol ratio; UHR, uric acid-to-high-density lipoprotein cholesterol ratio.

## DISCUSSION

Vitamin D deficiency is common in the young adult population due to inadequate dietary intake and low sunlight exposure in this age group. In our study, we found that MHR, one of the new inflammatory parameters, is an important indicator of vitamin D deficiency in young healthy adults.

Our study identified lower levels of HDL-C and higher levels of LDL-C in the groups with vitamin D deficiency compared with those with normal vitamin D levels. In contrast, we observed no significant differences between groups in triglyceride and TC parameters, consistent with previous studies (17). Dyslipidemia observed in vitamin D deficiency leads to the release of proinflammatory cytokines, thus creating a favorable environment for an inflammatory milieu. Furthermore, having high LDL-C and low HDL-C levels in young adulthood might contribute to the development of atherosclerosis (18). As they grow older, individuals in this group can face serious complications associated with thrombotic and inflammatory processes. Eliminating vitamin D deficiency among young adults is imperative for averting severe complications (19). The intricate connection between vitamin D deficiency and inflammation within this age demographic underscores this urgency.

Our study revealed significantly higher MHR in groups with vitamin D deficiency compared with those without vitamin D deficiency. Additionally, we found a notable inverse correlation between MHR and 25(OH)D levels. In regression analysis, we found that increased MHR was an independent risk factor and predictor of vitamin D deficiency in the young adult population. Tang et al., in a study on 538 health care workers, reported that MHR and 25(OH)D levels were inversely correlated and increased MHR was a risk factor for vitamin D deficiency, which is consistent with our study results. Furthermore, it is noteworthy that 86.25% of the health care workers who participated in the study had vitamin D deficiency (20). In a study including 860 young adults, Mousa et al. found that MHR and 25(OH)D were inversely associated, which is also consistent with the results of our study (21). Compared with other novel inflammatory parameters in our study, MHR showed the most robust correlation with 25(OH)D levels. The lack of a pronounced correlation between NLR, PLR, MLR, and 25(OH)D levels might be due to the particular characteristics of our sample group. Our results can be attributed to the study on a cohort of healthy individuals in young adulthood.

Previous investigations have posited that HDL-C assumes a protective role against endothelial damage induced by LDL-C. Significantly, HDL-C exhibits the potential to curtail the pro-oxidant and proinflammatory attributes of monocytes, hindering the differentiation and proliferation of monocytes into macrophages (22). It is essential to note that HDL-C can curtail the proinflammatory attributes of monocytes, which can help prevent atherosclerosis. The resulting macrophages (derived from monocytes), give rise to foam cells that are indicative of atherosclerosis. Therefore, the efficacy of HDL-C in eliminating cholesterol residues from these macrophages presents antiinflammatory and antioxidant effects. As such, the effectiveness of HDL-C in removing cholesterol deposits from these macrophages provides antiinflammatory and antioxidant benefits (23). These observations suggest a plausible correlation between reduced levels of HDL-C, increased presence of monocytes, and inflammation. Hence, the MHR presents itself as a promising parameter for conditions with inflammation. The relationship between vitamin D and monocytes should also be considered when evaluating MHR regarding vitamin D deficiency. Vitamin D suppresses endoplasmic reticulum stress, downregulating adhesion molecules (*e.g.*, PSGL-1, β1-integrin, and β2-integrin), resulting in decreased monocyte activation. Additionally, it exerts an immunomodulatory effect by regulating monocyte inflammatory responses, attenuating cellular signaling, inhibiting proinflammatory gene activation, and preventing the release of cytokines, including the reduction of tumor necrosis factor (TNF)-α release and the inhibition of intracellular inflammatory pathways, such as p38 and the nuclear factor kappa B (NF-κB) pathway (24,25). Furthermore, a study of healthy individuals showed a significant downregulation of the vitamin D receptor in monocytes of individuals with vitamin D deficiency compared with those who had normal vitamin D levels. Expression of the vitamin D receptor was strongly associated with serum 25(OH)D levels. In addition, the same study found that the adhesion of monocytes to endothelial cells suggested an early stage of atherosclerosis that was higher in people with vitamin D deficiency (26). When we consider the effects of vitamin D on monocytes and HDL-C, MHR appears to be a crucial parameter for vitamin D deficiency, which was observed in our study. From a clinical perspective, the inclusion of MHR in routine health assessments may facilitate early detection of vitamin D deficiency and allow health care providers to intervene promptly with targeted interventions. Such interventions may include vitamin D supplementation, lifestyle changes, and patient education.

Our study showed that the UHR was significantly higher in groups with vitamin D deficiency compared with those without vitamin D deficiency. Additionally, we identified a significant inverse correlation between UHR levels and 25(OH)D levels. With regression analysis, we found that UHR was an independent risk factor and predictor of vitamin D deficiency in the young adult population. Uric acid, an end product of purine metabolism, exhibits an association with exacerbated metabolic conditions and kidney disease at elevated serum levels (27). Vitamin D deficiency is recognized for its potential to elevate uric acid levels, often attributed to an inflamed environment (28). The combination of uric acid and HDL-C as metabolic markers constitutes the UHR, which allows for a better prediction of metabolic deterioration and inflammatory processes (29). Furthermore, a combined assessment of uric acid and HDL-C as parameters in vitamin D deficiency in the young adult population has yet to be explored in the literature. Our results suggest that UHR might be a crucial parameter for understanding vitamin D deficiency in the young adult population.

The cross-sectional design was a limitation of our study. If we consider inflammation a protracted process, we could have drawn more accurate conclusions if we had observed the individuals over time. Additionally, considering that smoking influences NLR, RDW, mean platelet volume (MPV), and PLR, the exclusion of smokers from the study might have yielded more precise results (30,31). More comprehensive studies are needed to evaluate the relationship between vitamin D deficiency and changes in lipid profile.

In conclusion, our study suggests that MHR and UHR can be essential for predicting vitamin D deficiency in young adulthood. The Society of Endocrinology and Metabolism of Türkiye, the Endocrine Society, and the Brazilian Society of Endocrinology and Metabolism refrain from endorsing societal screening by measuring 25(OH)D levels in the general population despite the widespread prevalence of vitamin D deficiency (16,32,33). Given the remarkable sensitivity and specificity of MHR in detecting vitamin D deficiency, it emerges as a viable candidate for use as a screening test. In addition, evaluating patients with increased MHR and UHR values regarding vitamin D deficiency might also be considered an appropriate approach. With this study, we hope to contribute to the awareness of prevalent vitamin D deficiency by examining new inflammatory parameters and lipid profiles in young adults. Furthermore, with this study we have provided innovative parameters suitable for assessing vitamin D deficiency in young adults.
